# The Prevalence of NAFLD and Fibrosis in Bariatric Surgery Patients and the Reliability of Noninvasive Diagnostic Methods

**DOI:** 10.1155/2020/5023157

**Published:** 2020-04-26

**Authors:** Maurizio Soresi, Daniela Cabibi, Rosaria V. Giglio, Stefania Martorana, Giuseppina Guercio, Rossana Porcasi, Antonino Terranova, Luigi A. Lazzaro, Maria R. Emma, Giuseppa Augello, Melchiorre Cervello, Gianni Pantuso, Giuseppe Montalto, Lydia Giannitrapani

**Affiliations:** ^1^Department of Health Promotion Sciences, Maternal and Infant Care, Internal Medicine and Medical Specialties (PROMISE), University of Palermo, Palermo, Italy; ^2^Department of Surgical Oncological and Oral Sciences, Division of General and Oncological Surgery, University of Palermo, Palermo, Italy; ^3^Institute for Biomedical Research and Innovation (IRIB), National Research Council, Palermo, Italy

## Abstract

**Background:**

Bariatric surgery patients have a higher prevalence of nonalcoholic fatty liver (NAFL) than the general population; however, its assessment and the accurate staging of fibrosis are often complicated because noninvasive tests are not very accurate in patients with morbid obesity, and liver biopsy cannot be performed as a routine exam. The aim of this study was to evaluate (A) the histological prevalence of NAFL, nonalcoholic steatohepatitis (NASH), and fibrosis in patients undergoing bariatric surgery; (B) the reliability of ultrasound (US) in diagnosing NAFL; and (C) the reliability of various fibrosis scoring systems for defining fibrosis.

**Methods:**

US and intraoperative liver biopsy results were reviewed in 57 bariatric surgery patients. NAFL, NASH, and fibrosis were diagnosed according to the Kleiner scoring system. US diagnosis of liver steatosis was based on the bright liver. Fibrosis scores used were (i) the BMI, AST/ALT Ratio, Diabetes (BARD) scoring system; (ii) the nonalcoholic fatty liver disease (NAFLD) fibrosis score; and (iii) the fibrosis-4 (FIB-4) index.

**Results:**

The prevalence of NAFL was 81%, NASH 61.4%, and fibrosis 94% (F3 5.7%, cirrhosis 2.8%). The sensitivity of US was 95%, specificity 50%, and likelihood ratio (LR+, LR-) 1.91 and 0.1. The reliability of fibrosis scores for *F* ≥ 2 were as follows: BARD score: sensitivity 46%, specificity 54%, and area under the receiver-operating characteristics (AUROC) curve 0.5; NAFLD score: sensitivity 30%, specificity 89%, and AUROC 0.5; and FIB-4: sensitivity 68%, specificity 67%, and AUROC 0.7.

**Conclusions:**

In bariatric surgery patients, the prevalence of NAFL was 81%, NASH 61.4%, and fibrosis 94%. US is able to rule out the presence of NAFL, while the commonly used scores may be inaccurate in defining fibrosis in patients with morbid obesity.

## 1. Introduction

Nonalcoholic fatty liver disease (NAFLD) is a very common chronic liver disease worldwide, and in Western countries, it is the most frequent type of liver disease [1–7]. Diagnosis is based on an abnormal accumulation of fat in more than 5% of hepatocytes at liver biopsy, in the absence of other causes such as alcohol abuse, viral infection, autoimmune, or drug-related liver diseases. Since being overweight, obesity (especially visceral), dyslipidemia, and diabetes mellitus are recognized as risk factors for NAFLD, it is considered a component of metabolic syndrome (MS) [7–9].

The term NAFLD covers a wide spectrum of diseases, ranging from simple steatosis (NAFL) to nonalcoholic steatohepatitis (NASH), in which steatosis is associated with ballooning degeneration of hepatocytes and diffuse lobular inflammation; this is the form that can evolve towards liver fibrosis of varying degrees of severity, up to cirrhosis and hepatocellular carcinoma (HCC) [1–6].

Morbid obesity is often defined as a body mass index (BMI) exceeding 35 kg/m^2^ combined with at least one obesity-associated disease, or a BMI exceeding 40 kg/m^2^ with or without obesity-associated diseases [10]. NAFLD occurs frequently in people with morbid obesity, and it has been reported in 80-90% of patients undergoing bariatric surgery. An estimated 20-47% of these patients have NASH, of which 8-12% progress to cirrhosis and an undefined percentage to HCC [8–10]. From these data, it is clear that NAFLD, in its various stages of evolution, can adversely affect the health and survival of these patients. Therefore, the accurate evaluation of the presence of steatosis and the correct staging of fibrosis are important for the accurate assessment of these subjects' prognoses.

The gold standard for diagnosing steatosis and fibrosis is liver biopsy, but this is not always feasible as, in most patients, liver disease is asymptomatic and often without laboratory indications [1]. Several noninvasive tests have been proposed for defining fibrosis [1], and for the diagnosis of fatty liver disease, ultrasound (US) is certainly a reliable method, well accepted by patients and sufficiently accurate [10–14]. However, for fibrosis, elastography, considered the most reliable method for diagnosing chronic liver disease, is not sufficiently accurate in people with morbid obesity. Consequently, a number of alternative scores have been proposed [1, 15]. The most commonly used of these are the BMI, AST/ALT Ratio, Diabetes (BARD) scoring system, the NAFLD fibrosis score, and the fibrosis-4 (FIB-4) index [1, 16–18].

The aim of our study was to retrospectively evaluate (A) the histological prevalence of liver steatosis and fibrosis in a population of patients undergoing bariatric surgery; (B) the reliability of US in the diagnosis of steatosis; (C) the reliability of the various fibrosis scores listed above for defining liver fibrosis.

## 2. Patients and Methods

### 2.1. Patient Selection

We retrospectively examined US liver images and the results of intraoperative liver biopsies in 57 patients undergoing bariatric surgery, consecutively enrolled between January 2015 and December 2018 at the Bariatric Surgery Unit of the University Hospital of Palermo, Italy. The study was conducted in accordance with the ethical standards laid down in the Helsinki Declaration of 1975 and its subsequent amendments and was approved by the Ethics Committee as a spontaneous study on 23 June 2014 (No. 7/2014). All patients gave their approval and signed informed consent.

Before surgery, all patients had been interviewed and examined during an outpatient visit. A standard questionnaire was used to investigate the presence of metabolic, cardiovascular, or liver diseases, use of any hepatotoxic drugs, and alcohol consumption.

The patients then underwent a physical examination in which weight (kg), height (m), and BMI were measured. Blood pressure was evaluated with the patient in a sitting position, and hypertension was diagnosed according to the WHO/ISH criteria [19]. A blood sample was also taken from patients the morning before surgery to measure levels of glucose, total cholesterol and HDL, triglycerides, aminotransferases (AST/ALT), and insulin.

Diagnosis of type-2 diabetes mellitus (T2DM), impaired fasting glycemia (IFG), and impaired glucose tolerance (IGT) was performed in accordance with the expert committee on criteria for the diagnosis and classification of diabetes mellitus [20]. Insulin resistance was calculated using total insulin and glucose levels, following the homeostasis model assessment (HOMA-R) [21]. Patients with elevated transaminase values were tested for anti-HCV, HBsAg, ANA, AMA, ASMA, and LKM1 antibodies.

US was performed by a single operator the morning before surgery, after 10 hours of fasting, using a Philips 5000 HDI US machine with a 2-5 MHz convex multifrequency probe. NAFLD diagnosis was based on the presence of the bright liver on US [7]. Patients were divided into NAFLD-US positives or negatives.

Serum fibrosis scores were obtained using the following methods:

1. BARD scoring system, which consists of three variables: BMI > 28, 1 point; AST/ALT ratio > 0.8, 2 points; and T2DM, 1 point. The score varies from 0 to 4 points. According to Harrison et al. [17], scores of 2-4 points are associated with F3 or F4 stages of fibrosis, with an odds ratio of 17.333 (95% Cl; 3.639–82.558) and a negative predictive value of 97%
2. NAFLD fibrosis score, established with the following formula: −1.675 + 0.037 − age (years) + 0.094 − BMI (kg/m^2^) + 1.13 × IFG/diabetes (yes = 1, no = 0) + 0.99 × AST/ALT ratio − 0.013 × platelet count (×10^9^/L) − 0.66 × albumin (g/dL). According to Angulo et al. [18], a score below -1.455 (low cut-off) excludes advanced fibrosis, while a score higher than 0.676 predicts advanced fibrosis. The scores between these values are defined as indeterminate
3. FIB-4 index: $\text{age}\times \text{AST }\left(\text{U}/\text{L}\right)/\text{platelets}\left(10^{9}/L/\sqrt{\text{ALT}\left(\text{U}/\text{L}\right)}\right.$ [16].

### 2.2. Liver Histology

Wedge or core liver biopsies to the depth of Glisson's capsule were obtained shortly after the abdominal cavity was opened and before manipulation of the liver.

Liver biopsies were routinely formalin-fixed and paraffin-embedded. Hematoxylin and eosin (H&E) and picrosirius staining were available for each case. Slides were read by one expert pathologist (D.C.) who was unaware of patient identities and histories. A minimum biopsy specimen length of 15 mm or the presence of at least 10 complete portal tracts was required.

The Kleiner scoring system was used as a protocol for the histological assessment of steatosis [22]. This system evaluates the degree of NAFLD by assigning separate scores for steatosis, hepatocellular ballooning, lobular inflammation, and stage of fibrosis. Fibrosis was assessed on the 5-point scale (0-4) suggested by Kleiner et al. [22], in which stages F0 = absence of fibrosis, F1 = perisinusoidal or periportal fibrosis, F2 = perisinusoidal and portal/periportal fibrosis, F3 = bridged fibrosis, and F4 = cirrhosis.

The presence of NASH was assessed using the steatosis, activity, and fibrosis (SAF) score [23].

All patients who had an etiologically well-defined liver disease were excluded from the study.

### 2.3. Statistical Analysis

Data were expressed as mean ± standard deviation (SD) or as median and range (min-max). The prevalence of steatosis and fibrosis, sensitivity (Se), specificity (Sp), positive predictive value (PPV), negative predictive value (NPV), accuracy (Acc), and positive and negative likelihood ratio (LR+, LR-) were calculated using classical formulas. The receiver-operating characteristic (ROC) curve was constructed by calculating the sensitivity and specificity of individual serum fibrosis tests at different cut-off points, and the corresponding area under the curve (AUROC) was calculated to evaluate the diagnostic accuracy of the tests in differentiating F ≥ 2 [24]. The best cut-off value was calculated as the maximum LR obtained using the following formula: LR = probability of true positive + probability of true negative/probability of false positive + probability of false negative [25].

## 3. Results

**Figure 1:**
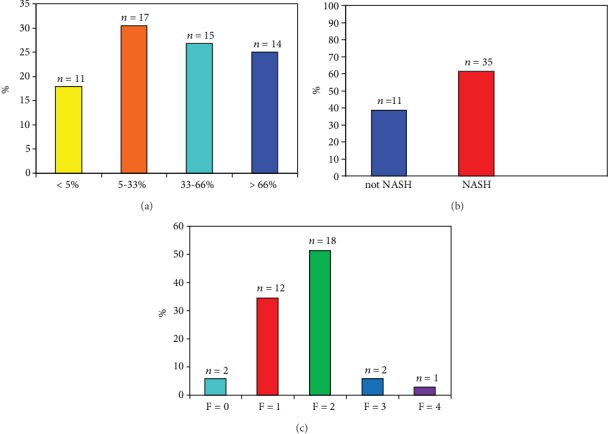
(a) Steatosis grading according to Kleiner. (b) Diagnosis of NAFLD according to SAF score. (c) Fibrosis score according to Kleiner.

Table 1 shows the clinical and demographic characteristics of the enrolled patients. Liver steatosis, defined as the accumulation of fatty acid in more than 5% of hepatocytes, was present in 46/57 biopsies (Figure 1(a)), with a prevalence of 81% (95% CI: 71-90). Figure 1(a) shows the steatosis grading according to the Kleiner scoring system. In particular, <5% (nonsteatosis, Grade 0) was found in 17.9% of biopsies; 5-33% (Grade I) in 30.4%; 33%-66% (Grade II) in 26.8%; and >66% (Grade III) in 25%.

Table 2 shows the diagnostic reliability data of US for steatosis diagnosis. Sensitivity was 95% (95% CI: 81-98), specificity 50% (95% CI: 18-75), PPV 89.4 (95% CI: 82-94), NPV 71% (95% CI: 36-92), accuracy 87% (95% CI: 75-94), positive LR 1.91 (95% CI: 1.1-3.5), and negative LR 0.1 (95% CI: 0.02-0.4).

Figure 1(b) shows the prevalence of NASH. NASH was present in 35/57 patients (61.4%; 95% CI: 58-73%). Of the 46 patients with steatosis, fibrosis was present in 40 (87%; 95% CI: 75-95). In patients with NASH, fibrosis was present in 33/35 (94%; 95% CI: 0.89-0.98) (Figure 1(c)), divided as follows: F0 fibrosis in 2 cases (5.7%; 95% CI: 1-30%), F1 in 12 cases (34.1%; 95% CI: 21-50%), F2 in 18 patients (51%; 95% CI: 18-35%), F3 (bridging fibrosis) in 2 patients (5.7%; 95% CI: 1-30%), and F4 (cirrhosis) in 1 case (2.8%; 95% CI: 0.5-20).

**Figure 2:**
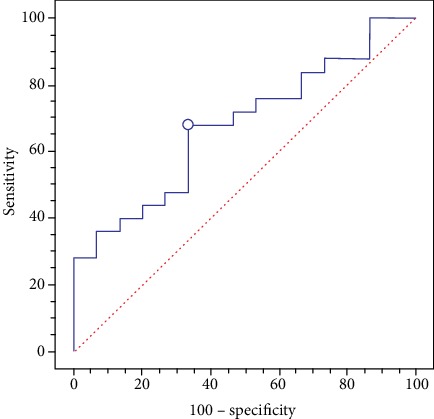
FIB-4 ROC curve with area under the curve of 0.7 and the best cut-off point.

The AUROC curves of the individual fibrosis tests and the best diagnostic cut-off values were calculated. The reliability of serum fibrosis scores in defining F ≥ 2 at the best cut-off values is shown in Table 3.  Figure 2 shows the AUROC curve of the FIB-4 index with an AUROC of 0.7 and the best cut-off point.

## 4. Discussion

Liver steatosis is considered the most frequent liver disease, especially in Western countries, and its prevalence is growing worldwide: 44% in the USA, 33% in Europe, and 25% in Italy. NAFLD is associated with obesity, dyslipidemia, and diabetes mellitus, and its prevalence varies according to the populations observed and the diagnostic methods used, including histology, US, elastography, or magnetic resonance imaging [1–7]. In patients with MS and dyslipidemia, a 78% prevalence of liver steatosis detected by US has been reported. In diabetics, the prevalence was between 30% and 80%, while in obese patients, it increased to between 81% and 87% [1–7].

NAFLD is associated with a higher mortality rate for cardiovascular, liver, and cancer diseases than the general population. In liver disease, the correct evaluation of a patient's histological evolution is crucial in defining prognosis [3]. The presence of fibrosis and its evolution towards cirrhosis negatively impact the survival of NAFLD patients.

The prevalence of steatosis in liver biopsies in our series was 81%, similar to the data reported in the literature. In contrast, the reliability of US in diagnosing steatosis in our study differed from values defined for the general population, where sensitivity has been estimated at 74%-91% and specificity at 85%-98%, depending on the case series considered [10–14]. In our study, sensitivity was comparable to data reported for the general population, whereas our 50% specificity was unsatisfactory. Literature data in the past have reported conflicting results for specificity in diagnosis by US [10, 26, 27], and our study confirmed these doubts. However, our data also highlighted a very interesting fact: LR- and LR+ analyses gave values of 0.1 and 1.9, respectively. In general, values of LR−≤0.1 in a diagnostic test indicate a great reliability in excluding the disease, whereas values of LR+>10 indicate that the test is able to indicate its presence with a high level of confidence. Based on these observations, our finding of LR−≤0.1 indicates that US has a high reliability in defining the absence of steatosis in obese subjects but that, with a LR+ of 1.9, it is not very reliable for determining its presence [28, 29].

The limited reliability of US in detecting steatosis in patients with morbid obesity, shown by the low specificity and low LR+, probably depends on a number of factors, mainly related to the physics of US diffusion. It is a known fact that the deeper US waves go, the greater their diffraction and the lower their resolution capacity. In short, the ability of US to define a correct echo pattern is reduced, and very often an incorrect increase in the regulation curve of the gain increases the echogenicity of the liver, producing false positives; the bright liver typical of steatosis is, in fact, hyperechoic.

Studies conducted on obese subjects have reported a 6-94% frequency of fibrosis and a 26-55% frequency of NASH [30–34]. In our series, according to the SAF score [23], NASH was present in 61.4% of patients. Our results are slightly higher than the average in the literature, likely due to various phenomena occurring in patients with morbid obesity, above all insulin resistance because of the greater degree and extent of inflammation. Since there is no reliable serum parameter for NASH, noninvasive parameters to define the presence of steatohepatitis [1] were not evaluated in our series.

The prevalence of fibrosis in all subjects with steatosis was 87%. In the 11 subjects with NAFL alone without inflammation, a fibrosis score of F1 was found. In NASH patients, the prevalence of fibrosis was 94% (95% CI: 0.89-0.98), distributed as follows: F1 in 12 cases (34.1%; 95% CI: 21-50%), F2 in 18 patients (51%; 95% CI: 18-35%), F3 (bridging fibrosis) in 2 patients (5.7%; 95% CI: 1-30%), and F4 (cirrhosis) in 1 case (2.8%; 95% CI: 0.5-20).

These data suggest several considerations. First, it is possible to find small amounts of fibrosis even in patients with NAFL alone, as reported in the literature. This may depend on many factors: the limits of biopsy sampling, or the possibility of finding a disease in phases where the presence of inflammation may be less accentuated. Second, despite the prevalence of fibrosis falling within the range reported by the literature, our percentage of advanced fibrosis (≥F3) was very low.

Although the validity of our considerations is limited by the small study population, this observation may also be due to the relatively young age of our patients (41 years). Age is indeed known to be one of the most important variables influencing NASH onset and evolution; in fact, as age increases, there is also an increased incidence of insulin resistance, MS, and diabetes mellitus [35, 36]. In a systematic review of this issue, Argo et al. found that age was the independent predictor of advanced fibrosis (HR 0.98, 95% CI: 0.96-0.99, *p* = 0.009) and that young people have a lower risk for the presence of fibrosis [37]. However, this study did not show any relationship between fibrosis and diabetes, in contrast to McPherson et al. who found an important role for diabetes among patients with NAFL: in fact, 80% of those having fibrosis progression were diabetic at the follow-up liver biopsy compared to 25% of nonprogressors (*p* = 0.005) [38].

Moreover, there is another reason which may explain the lower frequency of fibrosis in our study: the high prevalence of premenopausal women. Gender and reproductive states may influence the degree of fibrosis in patients with NASH. Longitudinal studies suggest that the incidence of NAFLD is higher in males than in females, and the incidence is higher in menopausal (7.5%)/postmenopausal (6.1%) women as compared to premenopausal (3.5%) women [39]. In addition, an Italian multicenter study found that men with NAFLD were approximately 10 years younger than women, confirming previous findings that premenopausal women are somehow “protected” from NAFLD [40].

Yang et al. found an adjusted cumulative odds ratio (ACOR) and 95% confidence interval (CI) for greater fibrosis severity of 1.4 (0.9, 2.1) (*p* = 0.17) for postmenopausal women, with premenopausal women as a reference [41].

Finally, Wang Z. et al., studying the predictive factors of NASH and fibrosis in women with different BMI and age, reported that the prevalence of NASH seems to be considerably higher in obese and postmenopausal women with NAFLD [42].

Further, the use of serum markers to evaluate the presence of fibrosis did not yield reliable results. The AUROC values of the BARD and NAFLD scores showed low accuracy. Only the FIB-4 index presented an acceptable ROC curve of 0.7, but the modest results obtained in our study were not enough to indicate it as being reliable. Our results are in disagreement with the literature, which recognizes an important role for the FIB-4 index and NAFLD score, but it is possible that the young age of our population played an important role in this result, given that the individual's age is included in the calculation of the scores. However, even in this case, some considerations must be made: (1) our limited number of cases may have reduced the reliability of the results, as shown by the wide confidence interval; (2) most of the patients reported in the guidelines did not suffer from morbid obesity [1]; (3) our patients showed a low degree of fibrosis; in fact, the accuracy of noninvasive fibrosis tests is known to be very limited for F ≤ 2 values [1]; (4) it possible that the young age of our patients also conditioned the results of the noninvasive fibrosis tests because most of the tools/calculators assessed take the age of the individual into account. Finally, we have to underline that our patients were all Italian, and therefore, our results cannot be generalized; in fact, both NASH and its evolution may vary according to ethnicity. For example, Hispanics with obesity and diabetes have a far higher risk for advanced liver disease than other racial or ethnic groups, indicating the potential involvement of other factors such as genetic variants [43].

## 5. Conclusions

In conclusion, our data show that US is a method that can exclude the presence of steatosis in subjects with severe obesity with a good degree of reliability.

Steatosis assessment with the controlled attenuation parameter using XL probes has recently been proposed in obese subjects and seems to have a good level of accuracy. It is advisable, in our opinion, to reserve this examination for patients in whom US images suggest steatosis, in order to confirm and eventually stage it.

The literature data report that, even in subjects with obesity, fibrosis serum markers are reliable; however, in our study, the accuracy of fibrosis evaluation with serum markers appeared to be limited, though this may be due to the low prevalence of fibrosis in our patients. In any case, the noninvasive evaluation of fibrosis in patients with morbid obesity is a chapter still to be written, as even elastosonographic methods have their limits. Although special XL probes, which seem to be able to reduce the need for nondiagnostic tests in subjects with severe obesity, have recently been used for morbidly obese patients, the values obtained are lower than those in nonobese subjects, and therefore, there are no well-defined cut-offs that can indicate the reliability of the method [15].

## Figures and Tables

**Table 1:** Clinical and demographic characteristics of the enrolled patients.

	*n* = 57
Age (y)	42 ± 12.0
M/F	16/41
BMI	43.5 ± 7.1
Diabetes	13 (14.3%)
Hypertension	17 (29.8%)
HOMA	4 (2.4-6.9)
AST (U/L)	18.5 (16-27)
Elevated AST (>40 U/L)	3 (5.26%)
ALT (U/L)	29 (20-39)
Elevated ALT (>40 U/L)	9 (15.8%)
Total cholesterol (mg/dL)	192 ± 28.7
Triglycerides (mg/dL)	113 (68-140)
HDL (mg/dL)	54 ± 14.0
LDL (mg/dL)	115.0 ± 27.2

**Table 2:** Diagnostic reliability of ultrasound in the diagnosis of steatosis in the study population.

	Ultrasound
Sensitivity	95% (95% CI: 84-99)
Specificity	50% (95% CI: 18-81)
Likelihood ratio +	1.91% (95% CI: 1.1-3.5)
Likelihood ratio -	0.1% (95% CI: 0.02-0.4)
Positive predictive value	89.4% (95% CI: 82-94)
Negative predictive value	71% (95% CI: 36-92)
Accuracy	87% (95% CI: 75-94)

**Table 3:** Reliability of serum markers in the definition of moderate fibrosis (F ≥ 2).

	NAFLD score	BARD score	FIB-4
	Cut − off<−1.455 % (95% CI)	Cut − off ≥ 2 % (95% CI)	Cut − off > 0.41 % (95% CI)
Sensitivity	30 (12-54)	46 (27-67)	68 (46-85)
Specificity	89 (52-99)	54 (27-79)	67 (52-98)
Likelihood ratio +	2.7 (1.3-5.5)	0.6 (0.1-3)	2 (1.3-3.2)
Likelihood ratio -	0.8 (0.1-5.1)	1.1 (0.84-1.34)	0.5 (0.1-4.4)
Positive predictive value	93 (82-97)	50 (13-86)	77 (45-92)
Negative predictive value	22 (12-38)	36 (31-42)	55 (29-78)
Accuracy	64.3 (48-78.5)	37 (23-54)	60 (42-74)
AUROC^∗^	0.53	0.5	0.7

^∗^The area under the receiver-operating characteristic.

## Data Availability

The data used to support the findings of this study are available from the corresponding author upon request.
